# Consumers and the Internet

**DOI:** 10.2196/jmir.1.suppl1.e3

**Published:** 1999-09-19

**Authors:** Alejandro Jadad

**Affiliations:** ^1^Co-Director, Canadian Cochrane CentreProfessor for Clinical Epidemiology and BiostatisticsMcMaster UniversityCanada

## Abstract

Although it is impossible to predict its evolution, recent developments and trends indicate that the Internet will have a profound effect on the role of patients and the general public in health care decisions and on how they interact with clinicians and other groups of decision-makers. The Internet is providing extraordinary opportunities to build strong partnerships between consumers and any other group involved in health care decisions. It has also the potential to create unprecedented division.

In this session, I will focus on key challenges that we must meet to develop optimal partnerships between consumers and other groups of decision-makers. Some of these include the need for:

- meaningful collaboration with consumers,
- efficient strategies to monitor patterns of Internet use among consumers,
- preparation for upcoming technological developments,
- balance between connectivity and privacy,
- better understanding of the balance between face-to-face and virtual interactions, and
- equitable access to technology and information across the globe.

The presentation is expected to motivate discussion and debate around issues that require immediate attention if we are to maximize the benefit of the Internet in health care.

